# Investigation of Crystallization, Morphology, and Mechanical Properties of Polypropylene/Polypropylene-Polyethylene Block Copolymer Blends

**DOI:** 10.3390/polym15244680

**Published:** 2023-12-12

**Authors:** Wenjun Shao, Li-Zhi Liu, Ying Wang, Yuanxia Wang, Ying Shi, Lixin Song

**Affiliations:** 1 Advanced Manufacturing Institute of Polymer Industry, Shenyang University of Chemical Technology, Shenyang 110142, China; shaowenjun0813@163.com (W.S.); violetlj@yahoo.com (L.-Z.L.); wy584436393@163.com (Y.W.); lxsong@syuct.edu.cn (L.S.); 2 School of Materials Science and Engineering, Shenyang University of Technology, Shenyang 110870, China; 3 Dongguan HAILI Chemical Material Co., Ltd., Dongguan 523808, China

**Keywords:** polypropylene, block copolymer, crystal structure, compatibility, X-ray

## Abstract

Polyethylene (PE)-based elastomers are the ideal choice for enhancing the compatibility of polypropylene/polyethylene (PP/PE) blends and improving the mechanical properties of PP-based materials. However, the issue of blend systems lies in the interplay between the crystallization processes. Therefore, we investigated the crystallization behavior during the cooling process of a new generation of PP/PE block copolymers (PP-b-PE) and random polypropylene (PPR, a copolymer of propylene and a small amount of ethylene or an alpha-olefin) blends using in-situ X-ray diffraction/scattering and differential scanning calorimetry (DSC) techniques. We also conducted mechanical performance tests on PPR/PP-b-PE blends at room temperature and low temperature (−5 °C). The results indicate that during the cooling process, the PP phase of PP-b-PE will follow the PPR to crystallize in advance and form a eutectic mixture, thereby enhancing the compatibility of PP/PE. Moreover, the PPR/PP-b-PE blend will form stable β-(300) crystals with excellent mechanical properties. Due to the improved compatibility of PP/PE with PP-b-PE, PE crystals are dispersed within PP crystals, providing bonding that improves the toughness of PPR under the low stiffness failure conditions of PPR/PP-b-PE blends, thereby enhancing their impact performance at low and room temperatures. This research has great significance for both recycling waste plastics and enhancing the low-temperature toughness of PPR.

## 1. Introduction

It is widely known that despite the similarity of their chemical structure, polyethylene (PE) and polypropylene (PP) are incompatible [1,2,3,4]. Random polypropylene (PPR) is a PP that modified polypropylene obtained by adding a small amount of ethylene or α-olefin. The short-chain distribution of PP and other comonomers in the PPR molecular chain is irregular. Compared with traditional PP, PPR has excellent physical and chemical properties. Therefore, PPR is widely used in areas such as water pipes. However, PPR pipes are prone to brittle cracking in low temperatures, which limits their application in cold areas, and the brittle nature of PPR at low temperatures is also a PP/PE compatibility issue [5]. Adding a compatibilizer is the most common approach [6,7,8,9,10]. Compatibilizers typically include block or graft polymers, or compatibilizers with the same, or miscible, monomer units as the blend components [11,12,13]. Block copolymers can reduce the phase separation behavior, and may exhibit a homogeneous or microphase separation morphology, becoming commonly used compatibilizers [14,15,16]. Block copolymers with alternating hard and soft segments can effectively improve the rough phase structure and poor mechanical properties of materials, making them important materials for industrial products and academic research [17,18,19].

Previous studies have been conducted on the application of block copolymers [20,21]. For example, Fortelny et al. [22] researched PP/PE/PS tri-block copolymers and found the interface to be the main factor affecting mechanical properties. Chaffin K et al. [23] investigated the mechanical properties of PP/PE blends and found that the toughness of semi-crystalline blends depends on the existence of crystallite-anchored interfacial entanglement and proposed that a small amount of block copolymer is needed for melt stabilization of PE/iPP blends. James M et al. [24] used catalysts to synthesize iPP/PE di-block and tetra-block copolymers, showing that the addition of block copolymers increases the elongation at break of PP/PE blends. The fact that block copolymers can form different phase separations determines the interface adhesion of the material and the macroscopic mechanical properties of polyolefin blends [25,26]. Ethylene octene block copolymers (OBCs) possess several characteristics, such as low octene content, high melting point, and rapid crystallization, which make them an ideal choice for improving the compatibility of PP/PE block copolymers [27,28]. Hence, some researchers use OBCs as compatibilizers to enhance the mechanical properties of PPR [7,29,30,31,32]. For instance, Qilin Ren significantly improved the toughness of PPR by blending OBCs and PPR [33]. However, since OBCs are PE-based block copolymers while PPR mainly consists of PP, there remain phase interface issues in OBC/PPR blends, resulting in a reduction in tensile strength. To overcome this problem, it is better to choose PP-based block copolymers. In 2013, Dow Chemical Company introduced a new PP-based block copolymer called INTUNE4544, which combines polyethylene, polyolefin elastomer (POE), and polar materials such as ethylene vinyl alcohol copolymer (EVOH) and polyamide (PA) with polypropylene. INTUNE4544 not only has the high impact resistance of elastomers but also avoids the phase interface problems caused by PE-based block copolymers. The investigation of PP-based block copolymers and the PPR blend on the structural properties of PPR is of great significance for improving the performance and optimizing the application of PPR [34,35]. On the other hand, while the brittle fracture problem of PPR has been extensively studied, its low-temperature brittleness as a pipe material remains unsolved, particularly in colder regions where pipes are exposed for prolonged periods. Consequently, improving the low-temperature brittleness of PPR remains of significant importance. Moreover, the mechanical properties of semi-crystalline polymers are dependent on their crystal structure. Therefore, understanding how changes in crystal structure affect mechanical properties is critical to obtaining high-performance materials [34]. Currently, research on block copolymers is limited, particularly with regards to how their crystal structure during cooling affects the material’s mechanical properties [35]. There is a lack of research on PP-based block copolymer modified PPR, and it is also important to have practical verification of the material that theoretically provides the most effective toughening of PPR. Real-time study of the crystalline structure of modified blends is crucial.

To address this issue, this article chose a new generation of PP-based block copolymers (PP-b-PE) to blend with PPR and investigate the effects of PP-b-PE on the mechanical properties of PPR at room temperature and low temperature (−5 °C). The changes in the crystal structure and interaction mechanism of PPR/PP-b-PE blends during cooling were studied in real-time using non-isothermal crystallization techniques (DSC) and synchrotron radiation X-ray (SAXS and WAXD). The relationship between the mechanical properties and crystal structure of PPR/PP-b-PE blends was explored through tensile and impact tests. The results of this research indicate that PP-b-PE can be utilized as a compatibilizer to enhance the compatibility of PP/PE and modify polyolefin materials. Furthermore, it has been demonstrated that PP-b-PE exhibits strong compatibility and interaction with PP-based polyolefin materials, even at low temperatures (−5 °C), which helps solve the low-temperature brittle cracking problem encountered in PPR.

## 2. Experimental

### 2.1. Preparation of Materials

The random polypropylene PPR503 from Sinopec Yangzi Petrochemical in Nanjing, China, and the block copolymer PP-b-PE, which is a combination of PP and PE produced by Dow in Delaware, USA, were used. The molecular weight (M_w_) of PP-b-PE is 16,000, and the mass ratio of the PP-b-PE PE chain to the PP chain is 49:51 [36]. PPR and PP-b-PE were melted together using a micro twin screw extrusion equipment (WGT200, Shanghai, China) at a temperature of 200 °C and blended according to the proportions listed in Table 1. The mixture was then granulated.

### 2.2. Thermal Analysis (DSC)

The thermodynamic behaviors of the blends were characterized using differential scanning calorimetry (TA, 200). The sample was initially heated from room temperature to 220 °C at a rate of 30 °C/min for 5 min to eliminate any thermal history of the crystal during processing. The sample was then cooled down to −30/20 °C at a rate of 5/10 °C/min and kept at this temperature for 3 min. Finally, the temperature was increased again to 200 °C at a rate of 10 °C/min.

### 2.3. Synchrotron Wide-Angle X-ray Diffraction (WAXD) Measurements

WAXD was conducted at the synchrotron radiation line station (BSRF 1W2A) at the Institute of High Energy Physics, Chinese Academy of Sciences, Beijing. For the WAXD experiments, the sample-to-detector distance was set at 89 mm and calibrated using aluminum oxide (Al_2_O_3_) as a standard. The detector used was a Mar 165 CCD (Mar USA Co., Palm Beach, FL, USA) with a light source wavelength of 1.54 Å and a recorded effective area of 165 mm. The resolution was 100 mm (FWHM), and the exposure time was 20 s. All 2D images were corrected for air scattering and beam fluctuations. Before any analysis, the collected data underwent air background correction. Data collection and processing were performed using the computer program “X-polar”.

An in situ temperature rising and falling WAXD experimental device was also used in this study. The sample was fixed on a heating table and heated to 200 °C at a rate of 30 °C/min, maintained at this temperature for 10 min, and then cooled down to 40 °C at a rate of 4 °C/min. During the cooling process, a 2D image was collected every 11 s. The collector used was a Mar 165 CCD, and the distance from the sample to the detector was set at 100 mm. The obtained 2D scattering diagram was processed using x-polar software (version 1.6.3.0) and integrated within the range of 135°–225° to obtain the WAXD cooling process spectrum.

### 2.4. Small Angle X-ray Scattering (SAXS)

SAXS was also conducted at the synchrotron radiation line station (BSRF 1W2A) at the Institute of High Energy Physics, Chinese Academy of Sciences, Beijing. The detector used was a Mar 165 CCD (USA) with a light source wavelength of 1.54 Å, an acquisition time of 20 s, a resolution of 100 mm (FWHM), and a sample-to-detector distance of 2900 mm. The data were collected using a Genix3D Cu operated at 50 kV and 0.6 mA (30 W). The detector area was (83.9 × 106.5) mm^2^, and the spot size was (0.8 × 0.8) mm^2^. The peak position q_max_ can be related to the long period L by Bragg’s law. Calculate the long period according to Formula (1). The collected data were corrected for air background prior to analysis using X-polar.

L = 2Π/q(1)

where q is the scattering vector defined as q = 4π (sin θ)/λ, where λ is the X-ray wavelength, θ is half the scattering angle (2θ), and L is the long period.

### 2.5. Mechanical Scattering

#### 2.5.1. Tensile Testing

A dumbbell-shaped sample rod with a length of 50 mm, neck length of 16 mm, and neck width of 4 mm was used to prepare samples with a thickness of 1 mm. The deformation test was conducted using an Instron 3365 tensile testing machine with a clamping distance of 20 mm. The specimens were stretched symmetrically at a constant strain rate of 100 mm/min at room temperature (25 °C), with the measuring point fixed in space. The average of five samples was collected for each material throughout the entire deformation process. All experimental data were averaged from five samples.

#### 2.5.2. Impact Test

The impact strength was measured using a s1107 according to GB/T 1843-2008 [37]. The maximum pendulum energy was 5.5 J (−5 °C) and 50 J (25 °C), and the impact speed was 3.5 m/s. The sample had a 2mm notch with a cross-sectional area of 4 mm^2^. The values obtained are presented as arithmetical means of the measurements on 10 specimens. All samples were kept at a constant temperature (−5 °C) for 24 h during the experiment.

### 2.6. Scanning Electron Microscopy (SEM)

Electron microscopy experiments were carried out with a high-resolution scanning electron microscope operated at 10 kV. All samples were gold sputtered prior to the SEM characterization. The Apreo-2C was used to observe the impact fracture surface and the morphological structure in the stress-whitened area of the PPR and PPR/PP-b-PE blend.

## 3. Results and Discussion

### 3.1. Non-Isothermal Crystallization Kinetics and Thermodynamic Behavior

**Figure 1:**
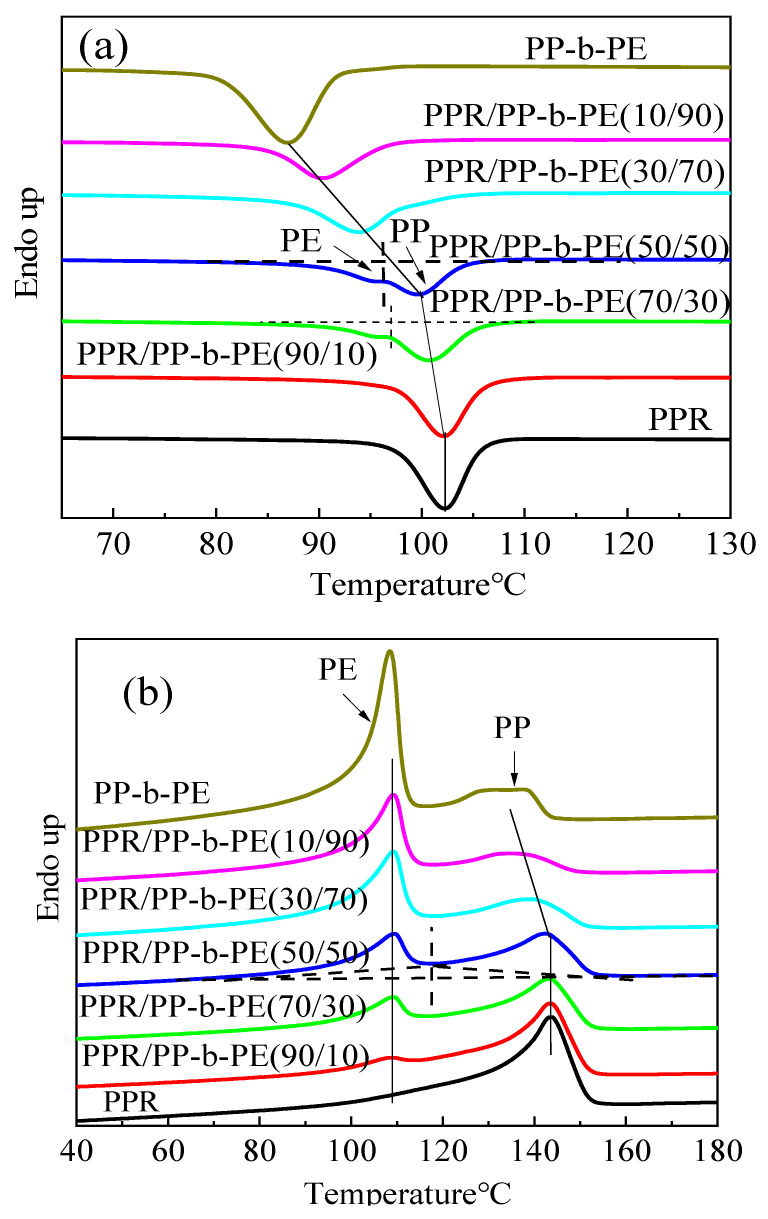
DSC curves of cooling (**a**) and heating (**b**) of PPR, PP-b-PE, and different proportions of PPR/PP-b-PE blends.

In Figure 1, the DSC results of the PPR/PP-b-PE blends are presented. Figure 1a shows the cooling curve of the PPR/PP-b-PE blends. The crystallization temperatures of pure PPR and the PPR/PP-b-PE (90/10) blend were 102.3 °C and 102.1 °C, respectively, with almost no change observed. Figure 1a indicates that PPR has an initial crystallization temperature of around 110 °C, whereas the initial crystallization temperature of PP-b-PE is approximately 91 °C, suggesting that PPR undergoes crystallization earlier than PP-b-PE. As PPR is essentially PP, it exhibits good compatibility with PP in PP-b-PE. The ratio of PP to PE in PP-b-PE is approximately 1:1 [36], and the PPR/PP-b-PE (90/10) blend contains 95 wt% of PP. The early crystallization of PPR in PPR/PP-b-PE (90/10) blends promote the early crystallization of PP (PP-b-PE). These findings indicate that a small amount of PP-b-PE has almost no effect on the non-isothermal crystallization in PPR/PP-b-PE blends.

When the content of PP-b-PE was further increased to 30–50 wt%, a small heat dissipation peak appeared at a lower temperature (as shown in Figure 1a), indicating that the PPR/PP-b-PE blend had a small microphase separation.

Further exploration of the crystallization process of phase separation was completed using PPR/PP-b-PE (50/50). According to the dashed line, the cooling curve and melting curve of the PPR/PP-b-PE (50/50) blend was divided into two parts, as shown in Figure 1. The crystallization and melting area ratios of PP peaks at high temperature and PE peaks at low temperature were calculated as 10:27 and 10:15, respectively. The results showed that the PP ratio of the PPR/PP-b-PE (50/50) blend in cooling crystallization was significantly higher than that in melting, indicating that both the PPR and PP phase from PP-b-PE contributed to the PP peak in cooling crystallization. This also showed that the PP segment in PP-b-PE had good compatibility with PPR and could co-crystallize. Since PPR has a higher crystallization temperature, PPR first crystallized in cooled crystallization and induced the PP phase (from PP-b-PE) to co-crystallize, thereby improving the compatibility of PPR and PP-b-PE. Additionally, the crystallization temperature of the PPR/PP-b-PE (10/90) blend was 102.1 °C, which was 15.2 °C higher than that of pure PP-b-PE. This suggests that a small amount of PPR (10 wt%) can also first crystallize and induce PP-b-PE to crystallize ahead of time. This happens because, as the content of PP-b-PE increases, the crystallization of PP-b-PE that is not induced by PPR to form nuclei becomes dominant, and a single peak is formed on the DSC cooling curve.

In Figure 1b, the second heating DSC traces of the PPR/PP-b-PE blends exhibit melting behavior. PP-b-PE has two melting peaks, of which the higher temperature is the wide melting peak of PP, which is platform-shaped, and the lower temperature is the sharp melting peak of PE. This is because PP-b-PE is a block copolymer of PP and PE. The molecular weight distribution of PP is wide and that of PE is narrow. PPR has only one melting peak at 143.8 °C. When the amount of PPR is 10 wt%, the crystallization temperature of the PE peak is 109.3 °C, which is higher than the Tm (PE) of PP-b-PE (108.5 °C). This is due to the early crystallization of PE induced by the first crystallization of PPR, forming a larger chain segment. However, when the PPR content increases, the melting point of PE almost does not change, because the PE of the generated larger sized chain segment is stable and does not change with the increase in PP-b-PE content. With an increase in PPR content, the melting peak of PP gradually narrows, and the strength gradually increases. The reason for the increased melting point is that PPR and PP-b-PE have good compatibility between the PPR and PP phases (from PP-b-PE), forming a relatively high molecular weight and uniform chain segment. Additionally, we conducted DSC testing while cooling down at a rate of 5 °C/min to −30 °C, and the results for Tg are summarized in Table 2. From Table 2, it can be observed that the Tg of PPR is −14.1 °C, while the Tg of PP-b-PE is −17.8 °C. As the content of PP-b-PE increases, the Tg gradually approaches that of PP-b-PE. Furthermore, the PPR/PP-b-PE blend exhibits only one Tg, which further indicates the good compatibility between PPR and PP-b-PE.

In summary, the addition of 10% PP-b-PE has no effect on the crystallization temperature of PPR. However, when the content exceeds 30%, PPR co-crystallizes with PP in PP-b-PE. This indicates that PP-b-PE has good compatibility with PPR. Moreover, even a small amount of PPR can induce early crystallization of PE in PP-b-PE.

### 3.2. In Situ Non-Isothermal Crystal Structure Change

**Figure 2:**
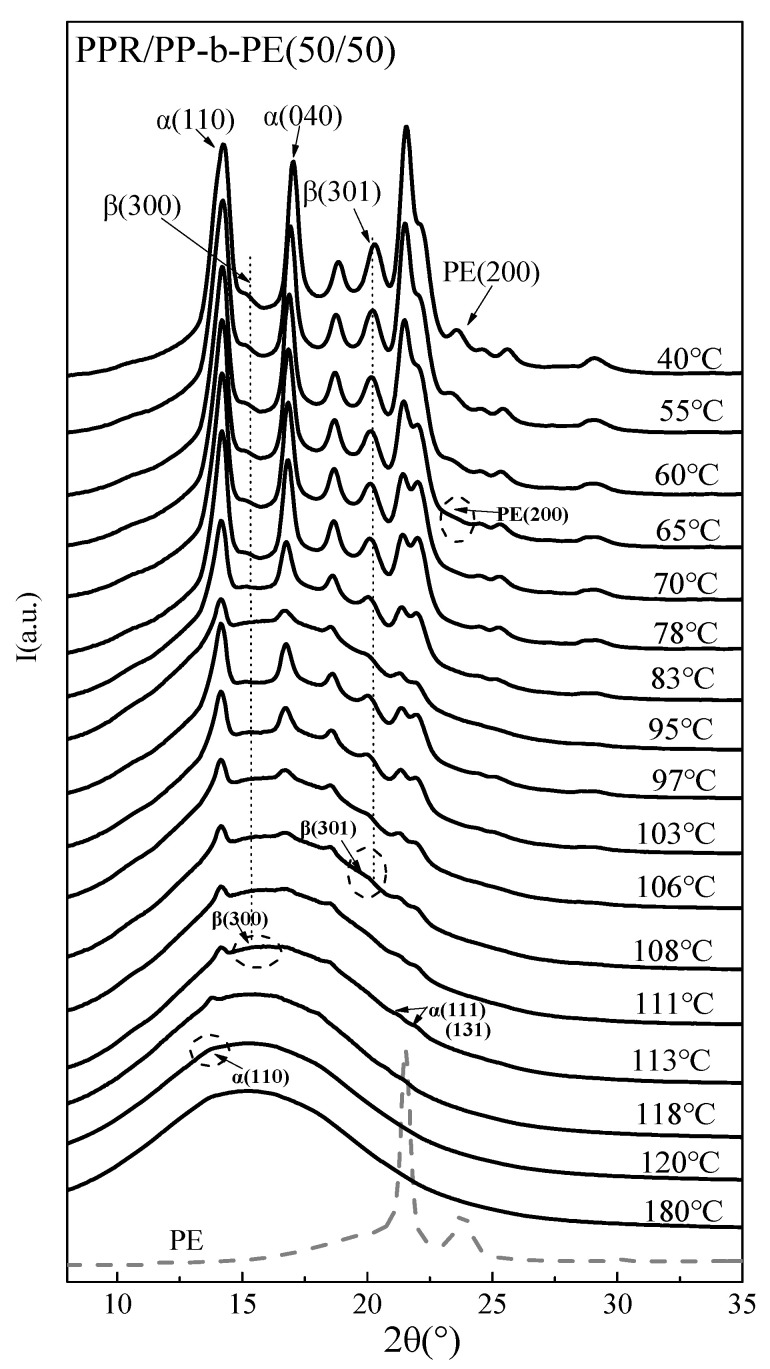
Linear WAXD profiles of PPR/PP-b-PE (50/50) blends cooling process at 4 °C/min.

**Figure 3:**
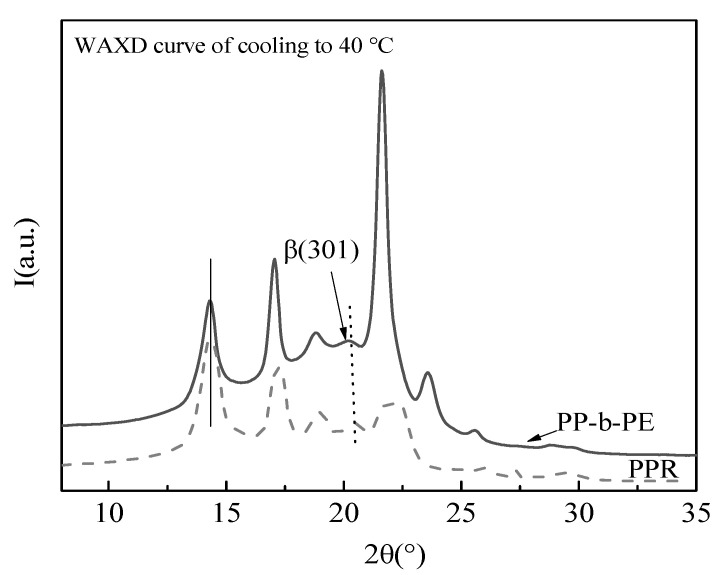
Linear WAXD profiles of PPR and PP-b-PE cooling to 40 °C at 4 °C/min.

The selected circularly averaged one-dimensional (1D) wide angle X-ray diffraction (WAXD) intensity profiles at various temperatures after cooling are shown in Figure 2. WAXD was used to analyze the crystal structure of PPR/PP-b-PE blends during the cooling process. This study focused on the crystallization process of the PPR/PP-b-PE (50/50) blend during cooling. The initial pattern (T = 180 °C) showed diffuse scattering without any crystal reflections due to a completely amorphous polymer melt. When the temperature was reduced to 120 °C, a lower diffraction peak appeared near 14.2°, which corresponds to the PP (110) crystal plane and has a starting crystallization temperature much higher than PP-b-PE (as shown in the DSC curve in Figure 1a). This may be due to the strong crystallization ability of PPR. Within the experimentally determined crystallization temperature range of 120 °C to 40 °C, the diffraction intensity of the (110) peak increased with increasing crystallization temperature. When the temperature dropped to 111 °C, other PP α typical diffusion peaks of crystals (2θ angles of 14.1°, 16.8°, and 18.6°), corresponding to the reflections of the (110), (040), and (130) crystal planes, respectively, were observed. However, when the temperature cooled to 113 °C, a small new diffraction peak of the β-crystal of the PP phase (shown in the dotted line circle in Figure 2) appeared at 16.1°, corresponding to the (300) crystal plane. According to the cooling WAXD curves of PPR and PP-b-PE (Figure 3), it can be observed that during cooling crystallization, PP-b-PE and PPR only had a lower diffraction peak at 2θ angles of 15.3° corresponding to the reflections of (300) crystal planes. The β-(300) characteristic plane diffraction peak was not observed in Figure 3. This indicates that the PPR/PP-b-PE blends induced the formation of new β-crystals. With the further decrease in temperature, the relative intensity of the (300) plane of the β-crystal was gradually covered by the diffraction relative intensity of other crystal planes. Because the contribution of β (300) is relatively small, the scattering intensity was reduced. When the temperature dropped to 108 °C, another tiny characteristic peak of the PP β-crystal could be seen at about 20.3°, corresponding to the (301) crystal plane of the β-crystal. The characteristic peak of the β-crystal generated by the blend became increasingly sharper with decreasing temperature. As shown in Figure 3, the β-(301) crystal plane generated by the blend was provided jointly by PPR and PP-b-PE, this also proves the existence of simultaneous crystallization behavior of PPR and PP-b-PE.

The crystal structures of PPR/PP-b-PE blends were analyzed by WAXD, which revealed the presence of β-crystals with excellent mechanical properties. Furthermore, the good compatibility between PPR and PP phases in PP-b-PE led to the early crystallization of PP-b-PE this may help enhance the mechanical properties of the blend, which will be discussed below. The co-crystallization of PPR and PP phases in PP-b-PE during the cooling process, as demonstrated by WAXD, indicates good compatibility between PPR/PP-b-PE components. This finding is consistent with the conclusion drawn from DSC analysis.

### 3.3. Microcrystals and Arrangement Structure of Different Blending Systems

**Figure 4:**
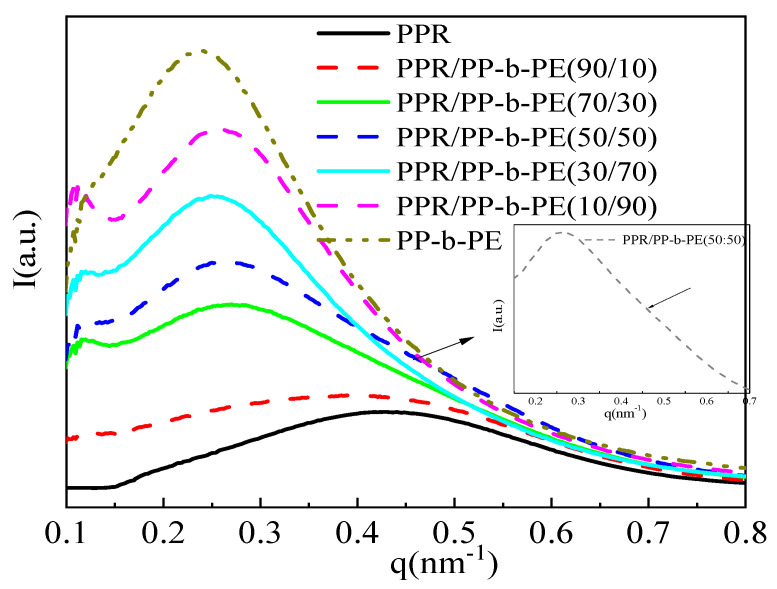
SAXS curves of PPR, PP-b-PE, and 10%, 30%, 50%, 70%, and 90% PPR/PP-b-PE blends.

Figure 4 displays the SAXS static I-q curve of the PPR/PP-b-PE blend at room temperature, revealing that the scattering intensity of PP-b-PE is significantly higher than that of PPR. According to the formula, the intensity of SAXS is proportional to the square of the difference in scattering length density (SLD) between two phases. The SLD difference between PE amorphous and crystal is larger than that of PP, the scattering intensity of PE is significantly higher than that of PP. Since PP-b-PE is a PP and PE block copolymer, it is composed of two polymers, PP and PE, with different SLDs, so PP-b-PE has the highest scattering intensity. On the other hand, pure PPR only has a SLD contrast between amorphous and crystalline PP, so PPR has the lowest scattering intensity. This explains the higher scattering intensity of PP-b-PE compared to PPR. The scattering intensity of the PPR/PP-b-PE (90/10) blend was only slightly increased compared with PPR, and the peak position remained almost unchanged. This was because most of the blend was made up of PPR, with only a small amount of PE phase added, resulting in no significant change to the crystal structure. As the PP-b-PE content increased, the peak intensity gradually decreased, and a small scattering peak appeared at a higher angle in the PPR/PP-b-PE (50/50) blend (as indicated by the arrow in Figure 4). This is due to the increase in PP content and the decrease in SLD contrast between PP and PE. When the content of PP-b-PE increased to 30 wt%, the scattering intensity increased significantly and the scattering peak showed an obvious move to a lower angle (q = 0.27), which may be due to the penetration of PP-b-PE into the amorphous region of PPR, resulting in an increase in crystal spacing. When the content of PP-b-PE continued to increase, there was only an increase in scattering intensity while the position remained almost unchanged. Based on the DSC results, this may be because PP-b-PE and PPR co-crystallize, but excessive PP-b-PE will crystallize separately. The crystal spacing comes from the average of all crystal spacing, so the change in scattering peak position is not significant.

In summary, the addition of 10 wt% PP-b-PE did not affect the crystal structure of PPR. However, when the content of PP-b-PE increased to 30–70 wt%, the excessive PP-b-PE penetrated the amorphous region of PPR, resulting in an increase in crystal spacing and a shift in the scattering peak position. Excessive PP-b-PE will crystallize separately, leading to a basically unchanged scattering peak position.

**Figure 5:**
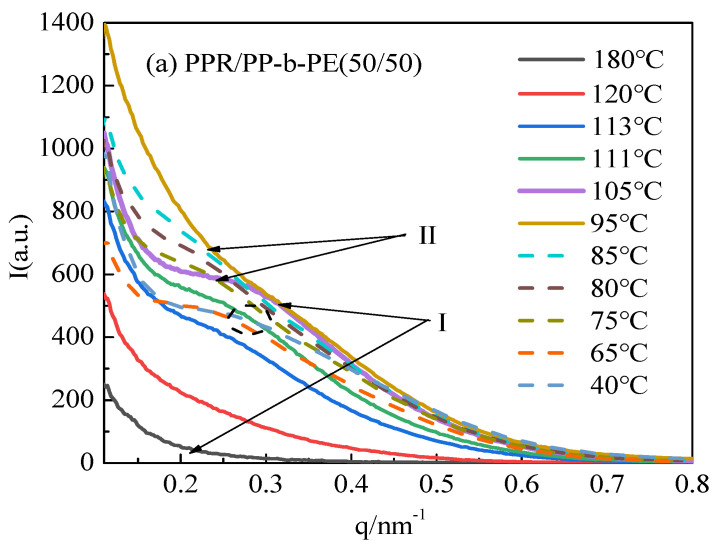

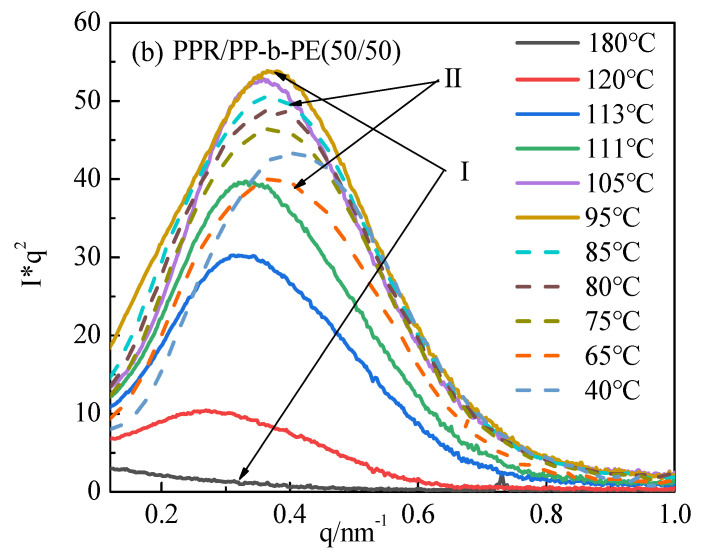
SAXS curves of PPR, PP-b-PE, and 10%, 30%, 50%, 70%, and 90% PPR/PP-b-PE blends (**a**) Linear-(I(q)-q) and (**b**) Lorentz-q^2^*I-(q).

The in situ SAXS technique has been widely used to investigate the effects of cooling on flow-induced crystallization, including the early stages and final microstructure. The ordered melt exhibits both short-range (unit lattice) and long-range (alternating crystal and chip structure) order. To further understand the microscopic crystal structure during cooling, we conducted an in situ SAXS experiment to study the cooling crystallization process. Figure 5 displays the SAXS curve (I(q)-q) of the PPR/PP-b-PE (50/50) blend. Figure 5a is divided into two regions based on intensity changes.

**Figure 6:**
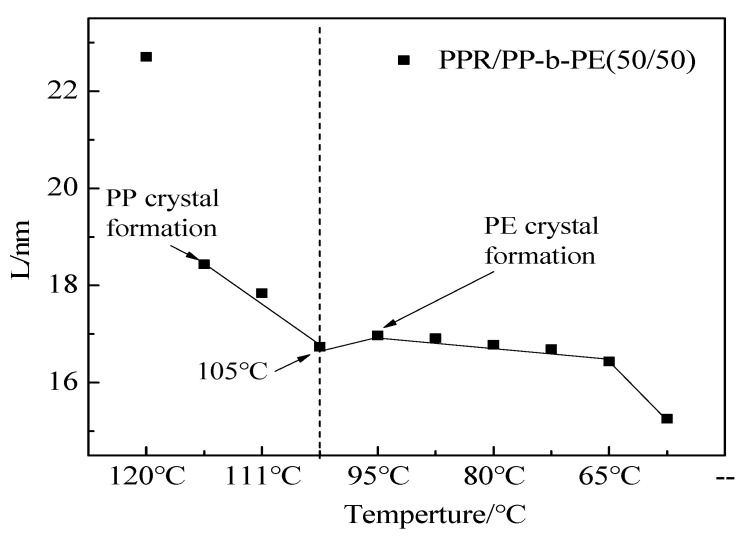
Long period curves of PPR/PP-b-PE (50/50) blends under difficult temperatures at 4 °C/min.

In region Ⅰ, when the temperature is 180 °C, the materials are in a completely molten state and the lamellar structure is destroyed. The system hardly contributes to the intensity of SAXS, and the curve does not show any characteristic peaks. As the temperature decreases from 180 °C to 95 °C, the scattering peak intensity increases while shifting towards the high vector direction, and the characteristics of the scattering peak become more apparent. At 113 °C, the characteristic crystal peaks can be observed, indicating the formation of PP phase crystals and the folding of PP chain segments, forming a long-range ordered periodic structure. Small lamella structures are more likely to undergo structural changes as the temperature decreases, resulting in significant changes in scattering intensity. The most distinct characteristic peak is observed at 105 °C, indicating that the crystal structure of PP is relatively perfect, forming a regular lamellar structure. The peak position of the maximum value in the scattering curve corresponds to the long period of the semi-crystalline polymer. The size of the polymer’s long period can be calculated using Formula (1). The Lorentz transformation method q^2^I-(q) is typically used to plot the scattering intensity q to accurately determine the peak position (as shown in Figure 5b). The results are displayed in Figure 6. It can be observed from Figure 6 that the long period structure of 18.5 nm appears at 113 °C, and the long period decreases to 16.7 nm when the temperature drops to 105 °C. This decrease is due to the orderly arrangement and tight packing of PP crystal chains.

In region Ⅱ, when the temperature is reduced to 95 °C, the scattering intensity reaches its maximum, but the characteristic peak disappears and only one peak shoulder appears. This phenomenon occurs because the PE phase crystal begins to form at 95 °C, resulting in a decrease in SLD contrast. On the other hand, the presence of PP crystals may affect the regularity of newly formed PE crystals and may also be one of the reasons this phenomenon occurs. As the temperature decreases further, the scattering intensity gradually decreases, and the peak position continues to shift towards the high vector direction. This decrease occurs because the scattering intensity of PE is significantly higher than that of PP. Due to the formation of PE crystals, the SLD becomes smaller, resulting in a reduction in scattering intensity. In addition, according to the DSC cooling curve (Figure 1), the PE crystal accounts for about 1/3 of the PP crystal, and although the proportion of crystal is small, the size of PE crystal is slightly larger than that of PP [38]. Therefore, after the formation of PE, it has little influence on the average size of the overall crystal, manifested as a small long-term change in the cooling region II.

The study examined the crystallization behavior of PPR/PP-b-PE (50/50) blends using in situ cooling experiments with SAXS. The results showed that PP crystallized first and formed small lamellar structures before the temperature decreased to 105 °C. As the temperature decreased, crystal compact accumulation and long period reduction occurred quickly. When the temperature reached 105 °C, PE began to crystallize, but its crystal regularity decreased, and the scattering intensity reduced during the process. Since the crystal size of PE was larger than that of PP, there was little long-term change observed.

### 3.4. Mechanical Behavior

The effects of PP-b-PE components on the mechanical properties of the PPR/PP-b-PE blend systems were investigated. Table 3 summarizes the modulus, yield stress, impact strength, breaking strength, and elongation at break values for the PPR/PP-b-PE blend. The elongation at break of PPR/PP-b-PE (70/30) blends increased by 14.2% compared to pure PPR (457.5%), reaching a value of 522.6%. The yield stress of PPR/PP-b-PE (70/30) (19.9 MPa) only decreased by 3.1 MPa compared to pure PPR (23.0 MPa). As the PP-b-PE content increased further, the elongation at break of the PPR/PP-b-PE blends continued to increase significantly, while stiffness only slightly decreased. These results demonstrate that the addition of PP-b-PE improves interface adhesion and indicates that PPR has good compatibility with PP-b-PE.

In addition to the tensile experiments, we also conducted impact tests on the blends, and the results are shown in Table 3. The increase in impact strength fell within the limits of experimental error. At room temperature (25 °C), when the amount of PP-b-PE added was 10–30 wt%, the impact strengths of PPR/PP-b-PE (90/10) (22.3 kJ/m^2^) and PPR/PP-b-PE (70/30) (58.0 kJ/m^2^) blends were 41.1% and 267.1% higher than that of pure PPR (15.8 kJ/m^2^), respectively. This shows that PP-b-PE has a significant toughening effect on PPR at room temperature. We also conducted impact experiments at the glass transition temperature of PP (−5 °C), and the results are also summarized in Table 3. The impact strength of PPR was found to be low at 4.1 kJ/m^2^. However, the impact strengths of PPR/PP-b-PE (90/10) and PPR/PP-b-PE (70/30) blends were significantly increased to 8.1 kJ/m^2^ and 9.8 kJ/m^2^, respectively, with an increase of 97.6% and 139%, respectively, compared to pure PPR. From Table 2, it can be seen that the Tg of the blended samples is all below −5 °C, and the Tg gradually decreases with the increase in PP-b-PE addition, which is also one of the reasons for the high impact strength. These results confirm that PP-b-PE also has a significant toughening effect on PPR at low temperature.

In conclusion, adding PP-b-PE can significantly increase the mechanical properties of PPR, owing to the good compatibility between PPR and the PP component of PP-b-PE, co-crystallization of PPR and part of PP-b-PE, and improved interface binding force. In blends, the phase interface is an important factor determining the material properties, including the mechanical properties, of the blends, thus improving the mechanical properties of PPR/PP-b-PE blends are essential. The better impact strength of PP-b-PE itself is also a contributing factor to this improvement. Additionally, the WAXD data analysis (Figure 4) indicates that PPR/PP-b-PE blends form β-crystals, which is another reason for the significant increase in the impact strength of PPR/PP-b-PE blends at low or room temperature.

In summary, studying the mechanical behavior of different samples has shown that the addition of PP-b-PE can significantly improve the impact strength and elongation at break of PPR at low and room temperatures without sacrificing stiffness. It is of great significance to toughen and improve the low-temperature brittleness of PPR.

### 3.5. SEM

**Figure 7:**
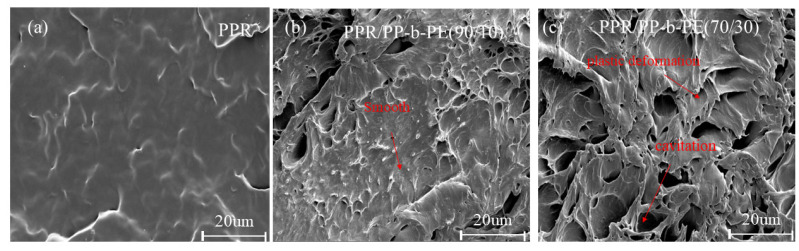
SEM images of the fracture surface of the PPR/PP-b-PE blend in specimens with different PP-b-PE content: (**a**) PPR (**b**) PPR/PP-b-PE (90/10); (**c**) PPR//PP-b-PE (90/10).

To better observe the toughening effect of PP-b-PE on PPR, we further studied the SEM images of the impact fracture surface of PPR/PP-b-PE blends. Since samples with a content of 50 wt% or higher of PP-b-PE did not fracture, we only studied the impact fracture surface SEM images of samples with 10–30 wt% PP-b-PE content. From Figure 7, there is a significant relationship between the morphology and composition of different blends. The impact fracture surface of pure PPR is relatively flat and clear, while the addition of only 10 wt% PP-b-PE results in a very rough impact fracture surface with localized voids and domain distortions. However, in Figure 7b, distinct relatively flat and clear areas can still be observed. As the content of PP-b-PE continues to increase, the impact fracture surface becomes rougher, with larger void sizes, and more pronounced plastic flow can be observed, along with fiberization and drawing caused by tough fractures. This further indicates that increasing the content of PP-b-PE enhances the impact strength of the blend (from 22.3 KJ/m^2^ to 58.0 KJ/m^2^). Additionally, from Figure 7c, distinct intertwining between the matrices can be observed. Impact fractures can be seen as the fractures of polymer molecular chains, and when the entanglement density is high, the impact fracture’s surface exhibits shear yielding as shown in Figure 7c, which requires a substantial amount of energy consumption. Therefore, adding 30 wt% PP-b-PE can increase the impact strength of the blend by 139% compared to PPR.

## 4. Conclusions and Discussion

The present study investigated the compatibility, crystal structure, and mechanical properties of new generation block copolymer PP-b-PE and PPR blends (hereafter referred to as PPR/PP-b-PE). Crystallization kinetics revealed that PPR crystallizes first during the cooling process, inducing early crystallization of PP-b-PE, which increases the crystallization temperature of PPR/PP-b-PE blends. When the PP-b-PE content exceeded 30 wt%, the PP crystallization peak of the PPR/PP-b-PE blend showed clear evidence of containing PP (from PP-b-PE) and PPR, indicating that PP-b-PaE and PPR have good compatibility.

The in situ non-isothermal WAXD/SAXS analysis identified a diffusion peak of the β-crystal in the PPR/PP-b-PE blends, with 2θ values of 16.1° and 20.8°, attributed to the crystal planes (300) and (301), respectively. The β-(301) crystal plane was supplied by PP-b-PE and PPR, while a new β-(300) crystal plane was generated, resulting in improved mechanical properties of the PPR/PP-b-PE blends. During cooling, PPR first crystallizes and induces early crystallization of PP-b-PE. As a result, the resulting PE is distributed in the PP crystal and plays a bonding role, enhancing the toughness of the PPR/PP-b-PE blends.

The mechanical properties test and SEM results demonstrated a significant increase in the impact strength of PPR/PP-b-PE blends, particularly when the PP-b-PE content was 30 wt%, resulting in increases of 267.1% (25 °C) and 139% (−5 °C), respectively. The impact strength also increased with the increase in PP-b-PE content. The addition of PP-b-PE can significantly increase the elongation at break of PPR/PP-b-PE blends at low and room temperature, with minimal damage to stiffness. These enhancements in mechanical properties indicate that PP-b-PE has good compatibility with PPR, which is crucial for addressing the compatibility problem between PP and PE and for the application of PPR tubes at low temperatures.

## Figures and Tables

**Table 1:** Samples with different proportions.

	Sample	PPR	PP-b-PE
1	PPR	100	0
2	PPR/PP-b-PE (90/10)	90	10
3	PPR/PP-b-PE (70/30)	70	30
4	PPR/PP-b-PE (50/50)	50	50
5	PPR/PP-b-PE (30/70)	30	70
6	PPR/PP-b-PE (10/90)	10	90
7	PP-b-PE	0	100

**Table 2:** Melting point and crystallization temperature of different samples.

Sample	Tc/°C	Tm (PE)/°C	Tm (PP)/°C	Tg/°C
PP-b-PE	86.9	108.5		−17.5
PPR/PP-b-PE (10/90)	90.6	109.3	136.5	−17.5
PPR/PP-b-PE (30/70)	93.9	109.1	140.0	−17.4
PPR/PP-b-PE (50/50)	99.6	109.1	142.8	−15.7
PPR/PP-b-PE (70/30)	100.7	108.9	143.3	−14.7
PPR/PP-b-PE (90/10)	102.1	107.8	143.4	−14.1
PPR	102.3	-	143.8	−13.8

**Table 3:** Mechanical data of samples.

Sample	Yield Stress	Elongation at Break	Impact Strength (KJ/m^2^)		Breaking Strength	Modulus
	MPa	(%)	25 °C	−5 °C	MPa	GPa
PPR	23.0	457.5	15.8	4.1	32.7	7.2
PPR/PP-b-PE (90/10)	21.1	467.1	22.3	8.1	31.3	6.5
PPR/PP-b-PE (70/30)	19.9	522.6	58.0	9.8	33.6	6.2
PPR/PP-b-PE (50/50)	16.5	535.1	unbreak		28.4	4.8
PPR/PP-b-PE (30/70)	13.9	573.5	unbreak		26.0	3.9
PPR/PP-b-PE (10/90)	12.5	694.4	unbreak		22.1	3.3
PP-b-PE	13.1	739.5	unbreak		25.6	3.7

## Data Availability

All the data are available within this manuscript.

## References

[1] Nedkov T., Lednický F. (2010). Morphologies of polyethylene-ethylene/propylene/diene monomer particles in polypropylene-rich polyolefin blends: Flake structure. J. Appl. Polym. Sci..

[2] Nedkov T., Lednicky F., Mihailova M. (2010). Compatibilization of PP/PE blends and scraps with Royalene: Mechanical properties, SAXS, and WAXS. J. Appl. Polym. Sci..

[3] Heino M., Kirjava J., Hietaoja P. (2015). Compatibilization of polyethylene terephthalate/polypropylene blends with styrene-ethylene/butylene-styrene (SEBS) block copolymers. J. Appl. Polym. Sci..

[4] Nakamura S., Tokumitsu K., Kitamura M., Miyagawa E., Tanaka A. (2007). A Study for Improvement of Mechanical Properties of Recycle PE/PP Blends. Resour. Process..

[5] Wu Y., Ge Q., Yang F., Wu T., Xiang M. (2021). Improving the low-temperature toughness of PPR pipe by compounding with PERT. J. Polym. Res..

[6] Kim B.C., Hwang S.S., Lim K.Y., Yoon K.J. (2000). Toughening of PP/EPDM blend by compatibilization. J. Appl. Polym. Sci..

[7] Zhang Z., Yu F., Zhou N., Zhang H. (2015). Compatibilization by Olefin Block Copolymer (OBC) in Polypropylene/Ethylene-Propylene-Diene Terpolymer (PP/EPDM) Blends. J. Macromol. Sci. B.

[8] Ubonnut L., Thongyai S., Praserthdam P. (2010). Interfacial adhesion enhancement of polyethylene–polypropylene mixtures by adding synthesized diisocyanate compatibilizers. J. Appl. Polym. Sci..

[9] Silva-Vela A., Roudet F., Calderón N., Huanca-Zuñiga P., Tupayachy-Quispe D., Almirón J. (2022). Study of the Mechanical Properties of Polymer Composites Based on Polyolefins with the Addition of Rice Husk and Compatibilizer. Mater. Sci. Forum.

[10] Penava N.V., Rek V., Houra I.F. (2012). Effect of EPDM as a compatibilizer on mechanical properties and morphology of PP/LDPE blends. J. Elastomers Plast..

[11] Kotlar H.K., Gustafson C.-G., Børve K.L. (2000). Polypropylene-phenol formaldehyde-based compatibilizers. II. Application in PP/PA6 75/25 (wt/wt) blends. J. Appl. Polym. Sci..

[12] Zhang Y., He J., Liu F. (2021). Synthesis of novel polycarbonate-based thermoplastic polyurethane elastomers compatibilizers with octadecyl side chains and their application in PC/PP blends. Polym. Adv. Technol..

[13] Lee B.Y., Lee D.H., Jang K.S. (2021). Impact Modifiers and Compatibilizers for Versatile Epoxy-Based Adhesive Films with Curing and Deoxidizing Capabilities. Polymers.

[14] Marić M., Macosko C.W. (2002). Block copolymer compatibilizers for polystyrene/poly(dimethylsiloxane) blends. J. Polym. Sci. Polym. Phys..

[15] Patel R., Park J.T., Hong H.P., Kim J.H., Min B.R. (2011). Use of block copolymer as compatibilizer in polyimide/zeolite composite membranes. Polym. Adv. Technol..

[16] Auriemma F., De Rosa C., Scoti M., Di Girolamo R., Malafronte A., D’alterio M.C., Boggioni L., Losio S., Boccia A.C., Tritto I. (2019). Structure and Mechanical Properties of Ethylene/1-Octene Multiblock Copolymers from Chain Shuttling Technology. Macromolecules.

[17] Saito M., Ito K., Yokoyama H. (2021). Mechanical Properties of Ultrathin Polystyrene-*b*-Polybutadiene-*b*-Polystyrene Block Copolymer Films: Film Thickness-Dependent Young’s Modulus. Macromolecules.

[18] Karaagac E., Koch T., Archodoulaki V.-M. (2021). Choosing an Effective Compatibilizer for a Virgin HDPE Rich-HDPE/PP Model Blend. Polymers.

[19] Wang P., Gao S., Chen X., Yang L., Cao T., Fan B., Liu J., Hu X. (2022). Effect of PCL-b-PEG Oligomer Containing Ionic Elements on Phase Interfacial Properties and Aggregated Structure of PLA/PCL Blends. Macromol. Res..

[20] Ma Y., He A., Liu C. (2021). Crystallization kinetics, crystalline structures and properties of PB/PP blends regulated by poly(butene-block-propylene) copolymers. Polymer.

[21] Sun X., Kharbas H., Peng J., Turng L.-S. (2015). A novel method of producing lightweight microcellular injection molded parts with improved ductility and toughness. Polymer.

[22] Fortelný I., Michálková D., Kruliš Z. (2004). An efficient method of material recycling of municipal plastic waste-ScienceDirect. Polym. Degrad. Stab..

[23] Yang F., Pan L., Du H.Z., Ma Z., Li Y.S. (2020). Effect of Olefin-based Compatibilizers on the Formation of continuous Structure in Immiscible HDPE/iPP Blends. Chin. J. Polym. Sci..

[24] Eagan J.M., Xu J., Di Girolamo R., Thurber C.M., Macosko C.W., LaPointe A.M., Bates F.S., Coates G.W. (2017). Combining polyethylene and polypropylene: Enhanced performance with PE/iPP multiblock polymers. Science.

[25] Lin Y., Yakovleva V., Chen H., Hiltner A., Baer E. (2010). Comparison of olefin copolymers as compatibilizers for polypropylene and high-density polyethylene. J. Appl. Polym. Sci..

[26] Souza F.G., Soares B.G., Manjunath A., Somashekar R. (2006). Blends of styrene-butadiene-styrene tri-block copolymer/polyaniline-characterization by SAXS. Polymer.

[27] Zhu H.Y., Tian F., Li X.H., Qiu H.B., Wang J. (2019). Crystallization and Phase Behavior in Block Copolymer Solution: An in Situ Small Angle X-ray Scattering Study. Chin. J. Polym. Sci..

[28] Li S., Lv Y., Sheng J., Tian H., Ning N., Zhang L., Wu H., Tian M. (2017). Morphology development of POE/PP thermoplastic vulcanizates (TPVs) during dynamic vulcanization. Eur. Polym. J..

[29] Jordan A.M., Kim K., Soetrisno D., Hannah J., Bates F.S., Jaffer S.A., Lhost O., Macosko C.W. (2018). Role of Crystallization on Polyolefin Interfaces: An Improved Outlook for Polyolefin Blends. Macromolecules.

[30] Xu J., Eagan J.M., Kim S.-S., Pan S., Lee B., Klimovica K., Jin K., Lin T.-W., Howard M.J., Ellison C.J. (2018). Compatibilization of Isotactic Polypropylene (*i*PP) and High-Density Polyethylene (HDPE) with *i*PP–PE Multiblock Copolymers. Macromolecules.

[31] Unger T., Klocke L., Herrington K., Miethlinger J. (2020). Investigation of the rheological and mechanical behavior of Polypropylene/ultra-high molecular weight polyethylene compounds related to new online process control. Polym. Test..

[32] Ren Q., Zhang Q., Wang L., Yi J., Feng J. (2017). Synergistic Toughening Effect of Olefin Block Copolymer and Highly Effective β-Nucleating Agent on the Low-Temperature Toughness of Polypropylene Random Copolymer. Ind. Eng. Chem. Res..

[33] Lin Y., Marchand G.R., Hiltner A., Baer E. (2011). Adhesion of olefin block copolymers to polypropylene and high density polyethylene and their effectiveness as compatibilizers in blends. Polymer.

[34] Ren Q., Fan J., Zhang Q., Yi J., Feng J. (2016). Toughened polypropylene random copolymer with olefin block copolymer. Mater. Des..

[35] Karaagac E., Koch T., Archodoulaki V.M. (2020). The effect of PP contamination in recycled high-density polyethylene (rPE-HD) from post-consumer bottle waste and their compatibilization with olefin block copolymer (OBC). Waste Manag..

[36] Hua X., Cheng J., Wang Y., Liu L.-Z., Wang Y., Shi Y. (2023). Crystallization, structure and properties of PP-b-PE block copolymer. Polymer.

[37] Liu J., Liu J. (2019). Characterization of maleic anhydride/styrene melt-grafted random copolypropylene and its impact on crystallization and mechanical properties of isotactic polypropylene. Polym. Bull..

[38] Wang Y., Shi Y., Shao W., Ren Y., Dong W., Zhang F., Liu L.-Z. (2020). Crystallization, Structures, and Properties of Different Polyolefins with Similar Grafting Degree of Maleic Anhydride. Polymers.

